# Mediators between bereavement and somatic symptoms

**DOI:** 10.1186/1471-2296-13-59

**Published:** 2012-06-18

**Authors:** Barna Konkolÿ Thege, János Pilling, Zoltán Cserháti, Maria S Koppˆ

**Affiliations:** 1 Institute of Behavioral Sciences, Semmelweis University, Nagyvárad tér 4, Budapest, H-1089, Hungary

**Keywords:** Anxiety, Bereavement, Depression, Gender differences, Somatic symptoms

## Abstract

**Background:**

In our research we examined the frequency of somatic symptoms among bereaved (N = 185) and non-bereaved men and women in a national representative sample (N = 4041) and investigated the possible mediating factors between bereavement status and somatic symptoms.

**Methods:**

Somatic symptoms were measured by the Patient Health Questionnaire (PHQ-15), anxiety with a four-point anxiety rating scale, and depression with a nine-item shortened version of the Beck Depression Inventory.

**Results:**

Among the bereaved, somatic symptoms proved to be significantly more frequent in both genders when compared to the non-bereaved, as did anxiety and depression. On the multivariate level, the results show that both anxiety and depression proved to be a mediator between somatic symptoms and bereavement. The effect sizes indicated that for both genders, anxiety was a stronger predictor of somatic symptoms than depression.

**Conclusions:**

The results of our research indicate that somatic symptoms accompanying bereavement are not direct consequences of this state but they can be traced back to the associated anxiety and depression. These results draw attention to the need to recognize anxiety and depression looming in the background of somatic complaints in bereavement and to the importance of the dissemination of related information.

## Background

According to a British study, a typical general practitioner has about 20 patient deaths annually, and thus even more of his/her patients will become bereaved in each year [1]. During the grieving period, somatic symptoms (e.g. headache, chest pain, dizziness, constipation) are particularly frequent. As a result, several questionnaires measuring grief examine somatic reactions as an independent dimension of grief [2,3]. Seldom can somatic diseases be diagnosed in the background of these symptoms; in the majority of cases they can be considered as psychosomatic reactions to bereavement [4]. However, functional symptoms which last a long time and appear in great quantity may cause clinically significant suffering, as well as the impairment of social and work functions. In this case it is possible to make a diagnosis of somatoform disorder [5]. The increased frequency of somatoform disorders has been verified in numerous groups of grievers, including widows [6], parents who have lost a child [7], those who have lost a close relative in a natural disaster [8], and the close relatives of those who died of cancer [9]. However, there is no sufficient data about the factors that contribute to the development of somatoform disorders among the bereaved.

In our study we surveyed the frequency of somatic symptoms among bereaved people who had lost close relatives (parents or spouse) in the past year, since somatic symptoms are most intensive in this period. Somatoform disorders are frequently associated with depression and anxiety [10-12]; therefore, our objective was to examine to what extent these factors can contribute to the formation of somatic symptoms. Since gender differences play a significant role in the sphere of somatoform disorders (the majority of patients are female [13]), we carried out our examinations stratified by gender to find out whether identical or different factors mediate between bereavement and somatic symptoms among men and women.

## Methods

### Participants

The present analyses were based on cross-sectional data from the Hungarostudy Epidemiological Panel Survey (HEP) 2006, a follow-up of the Hungarostudy 2002 nation-wide representative survey [14]. The sample was drawn from the National Population Register. The baseline data collection took place in 2002 and involved 12,668 subjects, who were representative of the adult population of Hungary according to age, gender, and 150 sub-regions. Those participants of the study, who had given consent for the follow-up, were contacted by our interviewers once again in 2006. Not counting those who had died, rejected to answer or were not able to answer the questions (e.g. due to their illness), finally, 4041 persons filled out the questionnaire in 2006. Regarding gender, age, and regions, the sample – following the weighing of the data – proved to be representative of the adult Hungarian population. The sampling methods are described in detail elsewhere [14,15]. The study was approved by the Ethics Committee of the Semmelweis University, Budapest.

Our sample included 185 people (76 males, 109 females) who had lost a close relative (spouse, mother or father) in the previous twelve months. Characteristics of the study sample are presented in Table 1.

**Table 1:** Characteristics of the sample stratified by gender and bereavement status

	**Males**		**Difference**	**Females**		**Difference**
	**Bereaved**	**Non-bereaved**		**Bereaved**	**Non-bereaved**	
N (%)	76	1501		109	2355	
	(4.8%)	(95.2%)		(4.4%)	(95.6%)	
Age						
mean	47	46	Z = −1.084	50	50	Z = −.189
SD	(14.11)	(17.14)	p = .278	(13.95)	(18.06)	p = .850
Educational level (%) basic	17	282	χ^2^ = 1.373	38	774	χ^2^ = 3.353
	(22.4%)	(18.8%)	p = .503	(35.2%)	(32.8%)	p = .187
secondary	51	1016		61	1213	
	(67.1%)	(67.7%)		(56.0%)	(51.5%)	
higher	8	203		10	368	
	(10.5%)	(13.5%)		(8.8%)	(15.6%)	
Anxiety						
mean	0.97	0.62		1.14	0.77	
SD	(1.06)	(0.78)	Z = −2.820	(0.95)	(0.88)	Z = −4.376
			p = .005			p < .001
Depression						
mean	12.37	7.64		11.77	9.32	
SD	(13.84)	(10.55)	Z = −3.206	(13.30)	(11.77)	Z = −2.662
			p = .001			p = .008
Somatic symptoms						
PHQ-15 mean	5.20	3.43		6.71	5.31	
SD	(6.00)	(3.84)		(5.87)	(4.97)	
minimal	45	1064		50	1313	
	(58.5%)	(70.9%)		(45.9%)	(55.7%)	
mild	21	312		30	624	
	(27.2%)	(20.8%)		(27.5%)	(26.5%)	
moderate	5	94		17	266	
	(6.2%)	(6.2%)		(15.6%)	(11.3%)	
severe^*^	6	31	Z = −2.471	12	152 (6.5)%	Z = −2.722
	(8.1%)	(2.1%)	p = .013	(11.0%)		p = .006

* minimal: 0–4, mild: 5–9, moderate: 10–14, severe: 15–30 [11].

### Measures and procedure

In addition to gender, age, educational level and bereavement status, variables measuring somatic symptoms, anxiety and depressive symptomatology were included in the analyses. Somatic symptoms were assessed by the Patient Health Questionnaire (PHQ-15) [16,17]. The PHQ-15 includes 15 symptoms (headache, stomach pain, chest pain, dizziness, etc.) that account for more than 90% of symptoms seen in primary care (exclusive of upper respiratory symptoms.) The PHQ-15 asks patients to rate how much they have been bothered by each symptom during the last month on a 0 (“not at all”) to 2 (“bothered a lot”) scale. Thus, the total score ranges from 0 to 30, with cut-off points of 5, 10 and 15 representing the thresholds for mild, moderate and severe somatic symptom severity. A score of ≥10 is the most commonly recommended cut-off point for clinically significant somatic symptom burden [14].

Anxiety was measured by the following question: “Have you been tense and nervous in the last two weeks?” The response categories were as follows: 0 = not at all, 1 = hardly ever, 2 = usually, 3 = definitely. Analysing the relationship of this question with the whole anxiety subscale of the HADS (Hospital Anxiety and Depression Scale [18]) on the 2002 sample of the HEP Survey, we found a very strong correlation between them (0.79; p < 0.001). Therefore, considering the large number of indicators in the HEP survey, only this single item was included in the 2006 wave of data collection. Depressive symptomatology was assessed by a nine-item shortened version [19] of the Beck Depression Inventory [20]. In this study, the internal reliability for this scale was very good (Cronbach’s alpha = .90).

### Statistical analyses

Statistical analyses were executed using the SPSS 20.0 software. At the bivariate level, the Mann–Whitney test and the Chi-square test were used to compare the data of bereaved and non-bereaved respondents. On the multivariate level, the general linear model procedure was used and the results were controlled for age and educational level. Effect size was expressed by partial eta-squared (η^2^). Throughout the analyses, data were stratified by gender.

## Results

On the bivariate level, we found that both bereaved men and women have significantly more somatic symptoms when compared to their non-bereaved counterparts. Altogether 14.3% of bereaved men reached a clinically significant score of 10 or more on the scale (8.4% of non-bereaved men), while in the case of bereaved women the percentage was 25.2% (17.8% of non-bereaved women). Depression and anxiety were significantly more frequent both among bereaved men (depression: p = .001, anxiety p = .005) and bereaved women (depression: p = .008, anxiety p = .000) (Table 1).

On the multivariate level, we tested first whether the loss of a close relative in the previous twelve months is associated with somatic symptoms even after controlling for our socio-demographic covariates. The data in our first model show that bereavement was also a significant predictor of PHQ scores for both genders in the multivariate analyses (Table 2). To examine the mediating role of anxiety, we added this variable to the second model and found that bereavement lost its role in predicting somatic symptoms regardless of gender. In contrast, when analysing the mediator function of depression (third model), our data revealed gender differences as well: when adding depression to the model, bereavement lost its predictive power in men but not in women. In the latter case depression proved to be only a partial mediator. Finally, to compare the predictive power of the two mediators, we added both variables to a fourth model. The results show that both anxiety and depression proved to be a significant predictor of somatic symptoms while bereavement did not – regardless of gender. Further, the effect sizes indicated that for both genders, anxiety was the stronger predictor of somatic symptoms.

**Table 2:** Results of the general linear model procedures predicting somatic symptoms

	**Males**				**Females**			
	**M sq.**	**F**	**p**	**η**^**2**^	**M sq.**	**F**	**p**	**η**^**2**^
*Model 1*								
Age	1516.0	103.2	<.001	.057	1262.6	52.4	<.001	.018
Educational level	343.8	23.4	<.001	.013	1153.6	47.9	<.001	.017
Bereavement status	117.1	8.0	.005	.005	207.0	8.6	.003	.003
Model	774.5	52.7	<.001	.084	1382.9	57.4	<.001	.058
*Model 2*								
Age	1454.0	124.1	<.001	.068	1141.0	61.7	<.001	.021
Educational level	287.3	24.5	<.001	.014	530.9	28.7	<.001	.010
Bereavement status	11.4	1.0	.323	.001	16.9	0.9	.339	.000
Anxiety	5019.4	428.5	<.001	.200	15859.9	857.9	<.001	.234
Model	1820.3	155.4	<.001	.266	4998.7	270.4	<.001	.278
*Model 3*								
Age	530.3	44.5	<.001	.025	32.3	1.7	.191	.001
Educational level	58.0	4.9	.027	.003	92.2	4.9	.027	.002
Bereavement status	20.9	1.8	.186	.001	94,0	5.0	.026	.002
Depression	4649.7	390.5	<.001	.186	14566.0	770.0	<.001	.216
Model	1727.7	145.1	<.001	.254	4675.0	247.1	<.001	.261
*Model 4*								
Age	796.9	72.0	<.001	.040	295.6	16.9	<.001	.006
Educational level	121.5	11.0	.001	.006	175.4	10.0	.002	.004
Bereavement status	6.1	0.6	.457	.000	25.2	1.4	.230	.001
Depression	1068.8	96.5	<.001	.054	2547.6	145.4	<.001	.049
Anxiety	1430.9	129.2	<.001	.070	3916.3	223.5	<.001	.074
Model	1668.4	150.6	<.001	.306	4523.3	258.1	<.001	.316

Note. M sq: Mean square; η^2^: partial eta-squared.

## Discussion

The results of our research indicate that somatic symptoms accompanying bereavement are not direct consequences of this state but they can be traced back to the associated anxiety and depression. Furthermore, our data revealed that anxiety was a mediator between bereavement and somatic symptoms in both genders, while depression played a mediating role only in men. These data draw attention to the need to recognize anxiety and depression looming in the background of somatic symptoms. Mental health problems may be masked by somatoform disorders [21]; therefore, clinicians need to be trained to recognize and treat more effectively the psychiatric disorders in the background of somatoform disorders [22].

Among the bereaved, this is the first research measuring individual somatic symptoms with the PHQ-15 questionnaire. The results confirm former studies which pointed out the co-morbidity of depression and anxiety [10-12]. Research in this field is all the more important since even today there is an ongoing professional debate about the criteria by which the diagnosis of “bereavement related disorder” may enter the DSM-V [23]. Psychosomatic symptoms are frequent and normal reactions during the grieving period, but clinicians should assess anxiety and depression underlying these phenomena. As previous studies have pointed out, the co-morbidity of somatoform symptoms, depression, and anxiety indicates an increased difficulty of coping with bereavement [24].

The strength of our study is that it is based on a national representative survey. According to a comprehensive study [12], only one analysis has been carried out using the PHQ-15 Questionnaire on a representative sample [25]. The limitation of the study, however, is its cross-sectional nature; thus it cannot verify causal relationships. Moreover, we analysed anxiety with only one question. Although the single question method used here is generally accepted and often used in large scale epidemiological surveys [26], and this question shows a very strong correlation with an often used and internationally recognised anxiety scale, the reliability of single item scales is inevitably weaker than that of longer scales. Thus we could have given a more reliably and nuanced picture of a person’s anxiety level with an extended and more complex instrument. Consequently, also the stronger mediator role of anxiety should be considered as preliminary result until confirmed in other studies using multi-item anxiety scales. It is also important to bear in mind that the PHQ-15 questionnaire gives no explanation of the causes of symptoms, which means that it is inappropriate for setting up a clinical diagnosis of somatization disorder. At the same time, the questionnaire had a sensitivity of 78% and a specificity of 71% for a DSM-IV somatoform diagnosis [27]. Finally, formation of somatic symptoms in bereavement can be affected by several other potential mediator factors (perceived social support, religious beliefs etc.) than anxiety and depression, which deserves further evaluation.

In conclusion, our research draws attention to the need to recognize anxiety and depression looming in the background of somatic disorders and provides useful data for setting up the diagnostic criteria of complicated grief.

## Competing interests

The authors declare that they have no competing interests.

## Authors’ contributions

BKT designed the study. JP and ZCs managed the literature searches. BKT conducted the statistical analysis. MK designed and managed the HEP 2006 research. JP wrote the first draft of the manuscript. All authors read and approved the final manuscript.

## Pre-publication history

The pre-publication history for this paper can be accessed here:

http://www.biomedcentral.com/1471-2296/13/59/prepub
